# Author Correction: Organizing principles of pulvino-cortical functional coupling in humans

**DOI:** 10.1038/s41467-019-09368-7

**Published:** 2019-03-26

**Authors:** Michael J. Arcaro, Mark A. Pinsk, Janice Chen, Sabine Kastner

**Affiliations:** 1 Princeton Neuroscience Institute, Princeton University, Princeton, NJ 08540 USA; 2 Department of Neurobiology, Harvard Medical School, Boston, MA 02115 USA; 3 Department of Psychological and Brain Sciences, Johns Hopkins University, Baltimore, MD 21218 USA; 4 Department of Psychology, Princeton University, Princeton, NJ 08540 USA

Correction to: *Nature Communications* 10.1038/s41467-018-07725-6, published online 19 December 2018

The original version of this Article contained an error in the author affiliations. Affiliation 3 incorrectly read ‘Department of Biological and Brain Sciences, Johns Hopkins University, Baltimore, MD 21218, USA’.

This has now been corrected in both the PDF and HTML versions of the Article.

