# Supplementary material for: Organizing principles of pulvino-cortical functional coupling in humans
Source: Nat Commun. 2018 Dec 19;9:5382. doi: 10.1038/s41467-018-07725-6 (PMC6300667; doi:10.1038/s41467-018-07725-6)
Supplement: Supplementary file 2 — Supplementary Information [file 41467_2018_7725_MOESM2_ESM.pdf]

Supplementary Note 1: Organization of the macaque pulvinar

The primate pulvinar is anatomically and functionally heterogeneous. It is sensitive to a variety of visual features [1, 2, 3] and cognitive states [4, 5, 6, 7, 8, 9, 10]. Traditionally, the pulvinar has been anatomically divided into lateral (PL), inferior (PI), medial (PM), and anterior (oral) regions in non-human primates [11], although more recent studies have identified finer segmentations within lateral and inferior regions [12, 13, 14, 15]. Inferior, lateral, and medial subdivisions of the pulvinar are interconnected with cortical regions involved in visual processing. The lateral pulvinar and lateral subdivisions of the inferior pulvinar are interconnected with occipital cortex [12, 16, 17]. Medial subdivisions of the inferior pulvinar are interconnected with several middle temporal areas including MT, MST, and FST [18, 19, 20, 21]. The medial pulvinar and dorsal sections of the lateral pulvinar are anatomically connected with parietal, frontal, and cingulate cortex [18, 22, 23].

Supplementary Note 2: Organization of cortical coupling in motion-sensitive cortex

Functional coupling of motion-sensitive cortical areas TO1/2 was localized to the ventral pulvinar (Figure 6b). While anatomical subregions could not be resolved within the human ventral pulvinar, the foci of these correlations were medial to the correlations of area V1 and anterior to ventral temporal areas, consistent with the location of medial subdivisions within the inferior pulvinar (PIcm, PIm, and Pip) that are anatomically interconnected with motion sensitive areas (MT complex) in monkeys [19, 24]. Interestingly, TO1/2 (possible homologue to monkey MT complex) were clustered with human occipital and temporal areas based on the network-level analyses of pulvino-cortical connectivity (Figure 2). The MT complex is traditionally associated with the dorsal stream [18, 25, 26]. However, comparable network-level analyses have not been performed based on pulvino-cortical connectivity in monkeys. A similar analysis based solely on cortico-cortical anatomical connectivity in macaques found a close link between MT and dorsal areas V3A and V3 [27] whereas an analysis of cortico-cortical functional coupling in humans found a closer link between TO1/2 and ventral stream areas LO1/2 [28]. Future work will be needed to resolve the relationship of MT to TO1/2 and their connectivity with the pulvinar across species.

The functional coupling between the pulvinar and TO1/2 appears to be situated within a broader topography linking lateral temporal regions and the ventral medial pulvinar. The foci of functional coupling for lateral temporal cortical regions, EBA and pSTS, were also found within medial portions of the ventral pulvinar, proximal to the TO foci. Though monkey homologues of these regions remain to be resolved, cortical regions surrounding MT in monkeys (e.g., FST and STP) are interconnected with parts of the pulvinar proximal to MT connections (i.e., adjacent to PIm) [18]. These data suggest that discrete pulvinocortical connections (such as between MT and PIm [19, 20, 21]) are embedded within a larger framework that preserves cortical topography [23].

Supplementary Note 3: - V1 cortical distance

To evaluate the influence of individual areas on the relationship between cortical distance and pulvino-cortical functional coupling, comparisons were made relative to a single reference area. V1 as the reference area (i.e., the distances between each area and V1) yielded the strongest correlation between cortical distance and the peaks of functional connectivity (Supplementary Figure 3a). To illustrate this relationship, we plotted the distances of peak correlations for all occipital-temporal areas (Supplementary Figure 3b) and for the subset of occipital, face-, and scene-selective ventral temporal areas (Supplementary Figure 3c) relative to V1. The distances for occipital-temporal areas fell close to a line between V1 and the maximally distant temporal area, AT.

Supplementary Methods:

Retinotopic Mapping. All subjects participated in a single scan session in which polar angle and eccentricity representations were measured across cortex using a standard traveling wave paradigm consisting of a wedge or annulus, respectively [29]. A subset of these subjects participated in two additional scan sessions in which polar

angle and eccentricity representations were measured within the pulvinar using a similar paradigm, but scanning protocols optimized for subcortical structures [30]. Stimuli mapped the central 15° of the visual field. Due to limitations of the scanner bore size and viewing angle, peripheral representations beyond 15° were not mapped nor included in any analyses. Each run consisted of eight 40s cycles. For each subject, 2-5 runs were collected for cortical mapping and 8-10 runs were collected for pulvinar mapping. Fourier analysis [31, 32, 33] was used to identify voxels that were sensitive to the spatial position (i.e., polar angle) of a peripheral cue during the task. Early visual and extrastriate areas V1, V2, V3, hV4, V3A–B, VO1–2, PHC1-2, LO1-2, TO1-2 were defined using standard criteria reported previously [32, 34, 35, 36, 37]. Pulvinar visual field maps, vPul1 and vPul2, and other subcortical visual field maps were defined using standard criteria previously published [30, 38, 39].

All subjects participated in a single scan session in which a memory-guided saccade task was used to localize topographically organized areas in parietal and frontal cortex [40, 41]. This task incorporates covert shifts of attention, spatial working memory, and saccadic eye movements in a traveling wave paradigm. The detailed description of the design and scanning parameters is provided in [40, 41]. Briefly, subjects had to remember and attend to the location of a peripheral cue over a delay period while maintaining central fixation. After the delay period, subjects had to execute a saccade to the remembered location and then immediately back to central fixation. The target cue was systematically moved on subsequent trials either clockwise or counterclockwise among eight equally spaced locations. Each run was composed of eight 40 s cycles of the eight target position sequence. A total of eight runs were collected in a single scan session for each subject. Fourier analysis [31, 32, 33] was used to identify voxels that were sensitive to the spatial position (i.e., polar angle) of a peripheral cue during the task. Parietal and frontal areas IPS0-5, SPL, FEF, and IFS were defined using criteria previously published [41, 42].

Object localizer. Sixteen subjects participated in a single scan session in which a standard object category localizer was used to define the occipital face area OFA; [43], fusiform face area FFA; [44, 45], anterior temporal face-selective area AT; [46]; the posterior superior temporal face area pSTS[46]; extrastriate body area EBA; [47], fusiform body area FBA; [48], parahippocampal place area PPA; [49, 50], transverse occipital sulcus TOS; [51], and retrosplenial cortex RSC; [52], and lateral occipital complex LOC[53]. Briefly, grayscale pictures of images (~12° x 12°) from five different categories (faces, headless bodies, inanimate objects, scrambled images, and scenes) were presented in 15s blocks, each containing 20 stimuli (350ms duration, 400ms interstimulus interval). Subjects viewed 12 blocks per stimulus category over the course of 4 runs. During stimulus presentation, subjects maintained central fixation and performed a one-back task indicating the repeated presentation of an object. Stimuli for each block were drawn from one of five categories: faces, scenes, headless bodies, intact generic objects, and scrambled pictures of generic objects. The OFA was defined as a region within the occipitotemporal sulcus that showed significantly stronger activity during the presentation of faces compared with intact object stimuli ($p < 0.0001$). The FFA was defined as a region within the lateral fusiform sulcus based on the same statistical criteria. For many subjects, this region included two distinct sub-regions in close anatomical proximity FFA-1/2; [46] pFus/mFus; [54]. AT was defined as a region within anterior temporal cortex based on the same contrast, though with a slightly lower threshold ($p < 0.01$). The pSTS was defined as a region within the posterior superior temporal sulcus that showed significantly stronger activity during the presentation of faces compared with intact object stimuli ($p < 0.0001$). The EBA was defined as a region within the lateral occipitotemporal cortex that showed significantly stronger activity during the presentation of headless bodies compared with intact object stimuli ($p < 0.0001$). The EBA partially overlapped retinotopic areas LO2 and TO1. The FBA was defined as a region within the fusiform sulcus based on the same statistical criteria. In several subjects, the FBA partially overlapped the FFA. Overlapping voxels were assigned to either the FFA or FBA based on contrasting activity during face and headless body presentations. The PPA was defined as a region within the posterior parahippocampal cortex within the collateral sulcus and along the medial fusiform sulcus that showed significantly stronger activity during the presentation of scenes compared with intact object stimuli

($p < 0.0001$). The PPA largely overlapped with retinotopic areas PHC1-2. Since the PPA was localized using a different experiment and may represent functional dissociations within this part of cortex, voxels were not restricted to non-overlapping portions. The TOS was defined as a region within the transverse occipital sulcus based on the same statistical criteria as the PPA. TOS partially overlapped with retinotopic areas V3B, IPS0, and LO1. The RSC was defined as a region within retrosplenial cortex based on the same statistical criteria as the PPA. LOC was defined as a region within the posterior superior temporal sulcus that showed significantly stronger activity during the presentation of intact objects compared with scrambled stimuli ($p < 0.0001$). Since the areas EBA, PPA, LOC, and TOS were localized separately from retinotopic mapping and may represent functional dissociations, voxels were not restricted to portions of cortex non-overlapping with retinotopic areas. Group-level regions were identified using a mixed effects meta-analysis (AFNI's 3dMEMA).

1. Maior RS, Hori E, Tomaz C, Ono T, Nishijo H. The monkey pulvinar neurons differentially respond to emotional expressions of human faces. *Behav Brain Res* **215**, 129-135 (2010).

2. Robinson DL, McClurkin JW, Kertzman C, Petersen SE. Visual responses of pulvinar and collicular neurons during eye movements of awake, trained macaques. *J Neurophysiol* **66**, 485-496 (1991).

3. Bender DB. Receptive-field properties of neurons in the macaque inferior pulvinar. *J Neurophysiol* **48**, 1-17 (1982).

4. Fischer J, Whitney D. Attention gates visual coding in the human pulvinar. *Nat Commun* **3**, 1051 (2012).

5. Hakamata Y*, et al.* The functional activity and effective connectivity of pulvinar are modulated by individual differences in threat-related attentional bias. *Sci Rep* **6**, 34777 (2016).

6. Komura Y, Nikkuni A, Hirashima N, Uetake T, Miyamoto A. Responses of pulvinar neurons reflect a subject's confidence in visual categorization. *Nat Neurosci* **16**, 749-755 (2013).

7. Saalmann YB, Pinsk MA, Wang L, Li X, Kastner S. The pulvinar regulates information transmission between cortical areas based on attention demands. *Science* **337**, 753-756 (2012).

8. Smith AT, Cotton PL, Bruno A, Moutsiana C. Dissociating vision and visual attention in the human pulvinar. *J Neurophysiol* **101**, 917-925 (2009).

9. Strumpf H*, et al.* The role of the pulvinar in distractor processing and visual search. *Hum Brain Mapp* **34**, 1115-1132 (2013).

10. Zhou H, Schafer RJ, Desimone R. Pulvinar-Cortex Interactions in Vision and Attention. *Neuron* **89**, 209-220 (2016).

11. Olszewski J. *The thalamus of the Macaca, mulatta: an atlas for use with the stereotaxic instrument.* Karger Publishers (1952).

12. Adams MM, Hof PR, Gattass R, Webster MJ, Ungerleider LG. Visual cortical projections and chemoarchitecture of macaque monkey pulvinar. *J Comp Neurol* **419**, 377-393 (2000).

13. Cola MG, Seltzer B, Preuss TM, Cusick CG. Neurochemical organization of chimpanzee inferior pulvinar complex. *J Comp Neurol* **484**, 299-312 (2005).

14. Gutierrez C, Yaun A, Cusick CG. Neurochemical subdivisions of the inferior pulvinar in macaque monkeys. *J Comp Neurol* **363**, 545-562 (1995).

15. Stepniewska I, Kaas JH. Architectonic subdivisions of the inferior pulvinar in New World and Old World monkeys. *Vis Neurosci* **14**, 1043-1060 (1997).

16. Gutierrez C, Cusick CG. Area V1 in macaque monkeys projects to multiple histochemically defined subdivisions of the inferior pulvinar complex. *Brain Res* **765**, 349-356 (1997).

17. Benevento LA, Davis B. Topographical projections of the prestriate cortex to the pulvinar nuclei in the macaque monkey: an autoradiographic study. *Exp Brain Res* **30**, 405-424 (1977).

18. Kaas JH, Lyon DC. Pulvinar contributions to the dorsal and ventral streams of visual processing in primates. *Brain Res Rev* **55**, 285-296 (2007).

19. Lin CS, Kaas JH. Projections from the medial nucleus of the inferior pulvinar complex to the middle temporal area of the visual cortex. *Neuroscience* **5**, 2219-2228 (1980).

20. Ungerleider LG, Desimone R, Galkin TW, Mishkin M. Subcortical projections of area MT in the macaque. *J Comp Neurol* **223**, 368-386 (1984).

21. Standage GP, Benevento LA. The organization of connections between the pulvinar and visual area MT in the macaque monkey. *Brain Res* **262**, 288-294 (1983).

22. Schmahmann JD, Pandya DN. Disconnection syndromes of basal ganglia, thalamus, and cerebrocerebellar systems. *Cortex* **44**, 1037-1066 (2008).

23. Shipp S. The functional logic of cortico-pulvinar connections. *Philos Trans R Soc Lond B Biol Sci* **358**, 1605-1624 (2003).

24. Kaas JH, Morel A. Connections of visual areas of the upper temporal lobe of owl monkeys: the MT crescent and dorsal and ventral subdivisions of FST. *J Neurosci* **13**, 534-546 (1993).

25. Ungerleider LG, Mishkin M. Two cortical visual systems. In: *Analysis of Visual Behavior* (ed^(eds Ingle DJ, Goodale MA, Mansfield RJW). MIT press (1982).

26. Goodale MA, Milner AD. Separate visual pathways for perception and action. *Trends Neurosci* **15**, 20-25 (1992).

27. Young MP. Objective analysis of the topological organization of the primate cortical visual system. *Nature* **358**, 152-155 (1992).

28. Haak KV, Beckmann CF. Objective analysis of the topological organization of the human cortical visual connectome suggests three visual pathways. *Cortex* **98**, 73-83 (2018).

29. Wang L, Mruczek RE, Arcaro MJ, Kastner S. Probabilistic Maps of Visual Topography in Human Cortex. *Cereb Cortex*, (2014).

30. Arcaro MJ, Pinsk MA, Kastner S. The Anatomical and Functional Organization of the Human Visual Pulvinar. *J Neurosci* **35**, 9848-9871 (2015).

31. Bandettini PA, Jesmanowicz A, Wong EC, Hyde JS. Processing strategies for time-course data sets in functional MRI of the human brain. *Magn Reson Med* **30**, 161-173 (1993).

32. Engel SA, Glover GH, Wandell BA. Retinotopic organization in human visual cortex and the spatial precision of functional MRI. *Cereb Cortex* **7**, 181-192 (1997).

33. Sereno MI, Pitzalis S, Martinez A. Mapping of contralateral space in retinotopic coordinates by a parietal cortical area in humans. *Science* **294**, 1350-1354 (2001).

34. Arcaro MJ, McMains SA, Singer BD, Kastner S. Retinotopic organization of human ventral visual cortex. *J Neurosci* **29**, 10638-10652 (2009).

35. Brewer AA, Liu J, Wade AR, Wandell BA. Visual field maps and stimulus selectivity in human ventral occipital cortex. *Nat Neurosci* **8**, 1102-1109 (2005).

36. DeYoe EA*, et al.* Mapping striate and extrastriate visual areas in human cerebral cortex. *Proc Natl Acad Sci U S A* **93**, 2382-2386 (1996).

37. Sereno MI*, et al.* Borders of multiple visual areas in humans revealed by functional magnetic resonance imaging. *Science* **268**, 889-893 (1995).

38. Schneider KA, Kastner S. Visual responses of the human superior colliculus: a high-resolution functional magnetic resonance imaging study. *Journal of neurophysiology* **94**, 2491-2503 (2005).

39. Schneider KA, Richter MC, Kastner S. Retinotopic organization and functional subdivisions of the human lateral geniculate nucleus: a high-resolution functional magnetic resonance imaging study. *J Neurosci* **24**, 8975-8985 (2004).

40. Konen CS, Kastner S. Representation of eye movements and stimulus motion in topographically organized areas of human posterior parietal cortex. *The Journal of neuroscience : the official journal of the Society for Neuroscience* **28**, 8361-8375 (2008).

41. Kastner S, DeSimone K, Konen CS, Szczepanski SM, Weiner KS, Schneider KA. Topographic maps in human frontal cortex revealed in memory-guided saccade and spatial working-memory tasks. *Journal of neurophysiology* **97**, 3494-3507 (2007).

42. Swisher JD, Halko MA, Merabet LB, McMains SA, Somers DC. Visual topography of human intraparietal sulcus. *The Journal of neuroscience : the official journal of the Society for Neuroscience* **27**, 5326-5337 (2007).

43. Puce A, Allison T, Asgari M, Gore JC, McCarthy G. Differential sensitivity of human visual cortex to faces, letterstrings, and textures: a functional magnetic resonance imaging study. *J Neurosci* **16**, 5205-5215 (1996).

44. Kanwisher N, McDermott J, Chun MM. The fusiform face area: a module in human extrastriate cortex specialized for face perception. *J Neurosci* **17**, 4302-4311 (1997).

45. McCarthy G, Puce A, Gore JC, Allison T. Face-specific processing in the human fusiform gyrus. *J Cogn Neurosci* **9**, 605-610 (1997).

46. Pinsk MA*, et al.* Neural representations of faces and body parts in macaque and human cortex: a comparative FMRI study. *J Neurophysiol* **101**, 2581-2600 (2009).

47. Downing PE, Jiang Y, Shuman M, Kanwisher N. A cortical area selective for visual processing of the human body. *Science* **293**, 2470-2473 (2001).

48. Peelen MV, Downing PE. Selectivity for the human body in the fusiform gyrus. *J Neurophysiol* **93**, 603-608 (2005).

49. Epstein R, Kanwisher N. A cortical representation of the local visual environment. *Nature* **392**, 598-601 (1998).

50. Aguirre GK, Detre JA, Alsop DC, D'Esposito M. The parahippocampus subserves topographical learning in man. *Cereb Cortex* **6**, 823-829 (1996).

51. Grill-Spector K. The neural basis of object perception. *Curr Opin Neurobiol* **13**, 159-166 (2003).

52. Maguire EA. The retrosplenial contribution to human navigation: a review of lesion and neuroimaging findings. *Scand J Psychol* **42**, 225-238 (2001).

53. Grill-Spector K, Kourtzi Z, Kanwisher N. The lateral occipital complex and its role in object recognition. *Vision Res* **41**, 1409-1422 (2001).

54. Grill-Spector K, Weiner KS. The functional architecture of the ventral temporal cortex and its role in categorization. *Nature reviews Neuroscience* **15**, 536-548 (2014).

Supplementary Figures

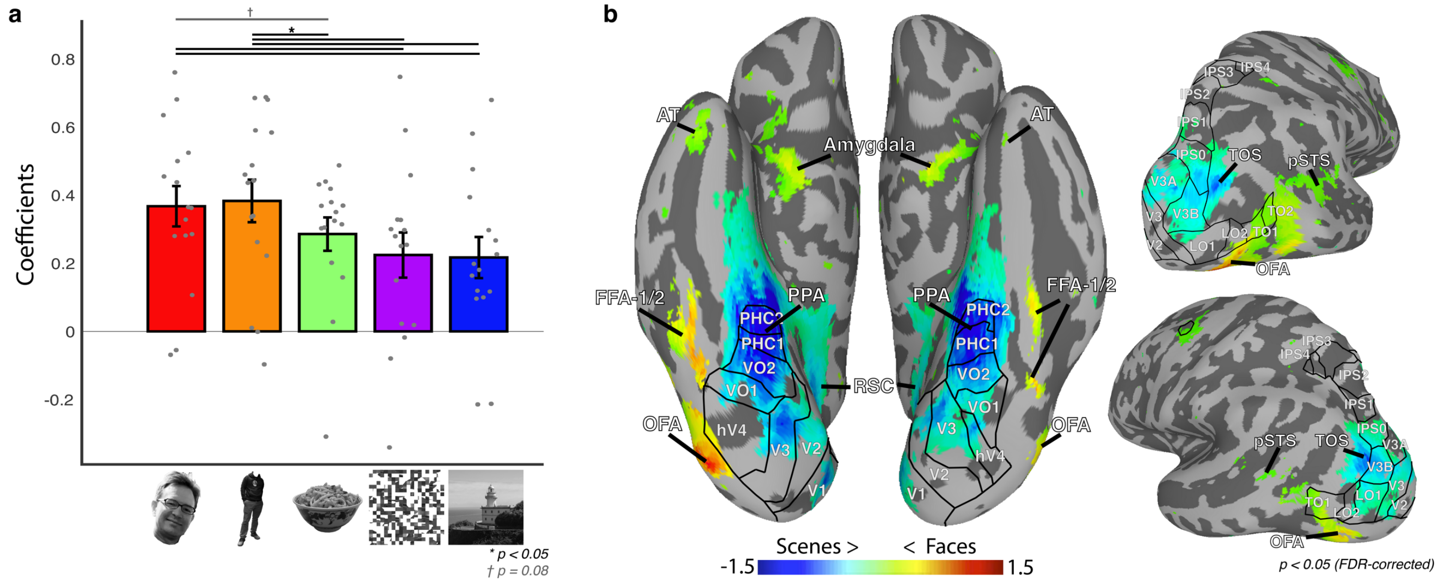

Supplemental Figure 1. Category localizer responses in the pulvinar and cortex. (a) Individual subject and group mean betas for face, headless body, object, scrambled, and scene categories in the posterior pulvinar. (b) Cortical activity for face vs. scene stimuli (p < 0.05, FDR-corrected, n = 16). Bar graphs show the group average and S.E.M for each category. Grey circles illustrate individual subjects.

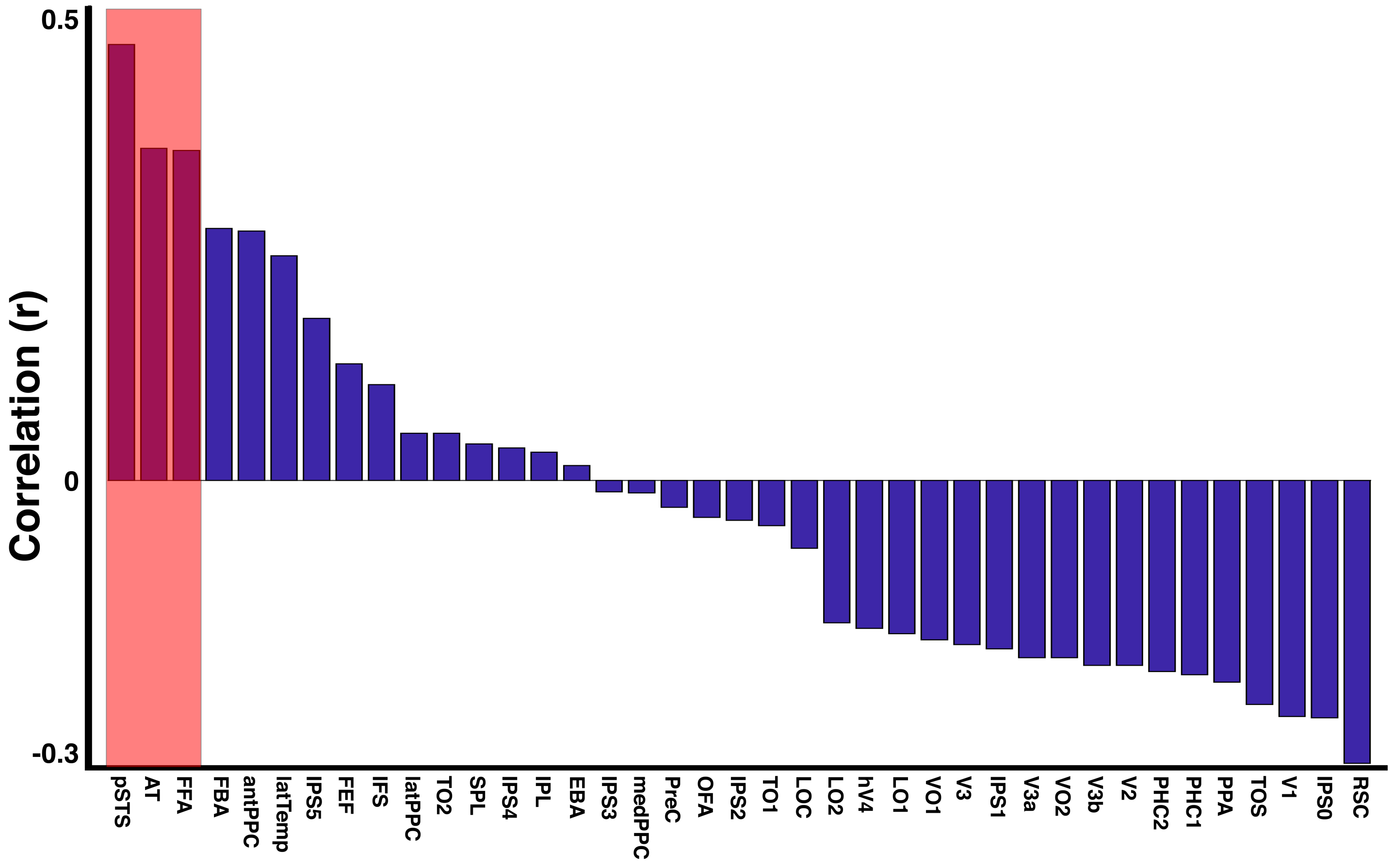

Supplementary Figure 2. Resting state pulvino-cortical coupling predicts functional tuning within the pulvinar. Correlation between the spatial pattern of face-minus-scene betas and each cortical areas funcitonal coupling within the pulvinar. The functional coupling at rest for face-selective cortical areas pSTS, AT, and FFA were most predictive of the face-minus-scene contrast within the pulvinar.

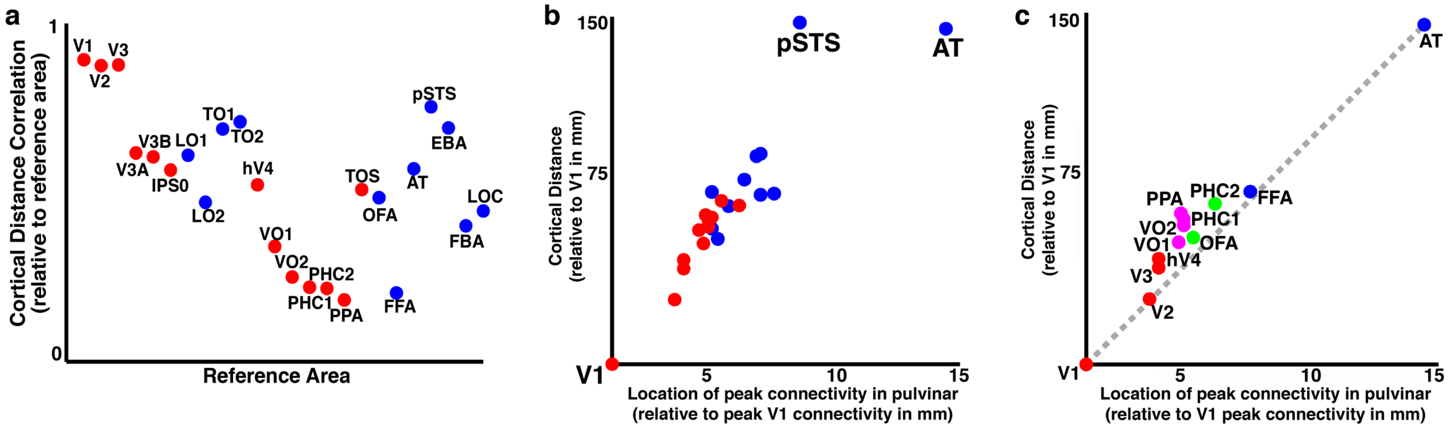

Supplementary Figure 3. Pulvino-cortical coupling reflects cortical distance from V1. (a) Graph shows the cortical distance correlation relative to each of the ventral areas. (b) A plot of the cortical distance relative to V1 vs. distance of peak location relative to V1 in the pulvinar for all 24 areas in Figure 6c. (c) Same plot as in (b), but for the subset of areas reported in Figure 6d.
